# Supplementary material for: Physiological and Transcriptome Responses of Pinus massoniana Seedlings Inoculated by Various Ecotypes of the Ectomycorrhizal Fungus Cenococcum geophilum during the Early Stage of Drought Stress
Source: J Fungi (Basel). 2024 Jan 15;10(1):71. doi: 10.3390/jof10010071 (PMC10817269; doi:10.3390/jof10010071)
Supplement: Supplementary file 1 [file jof-10-00071-s001.zip › jof-2787381-supplementary.pdf]

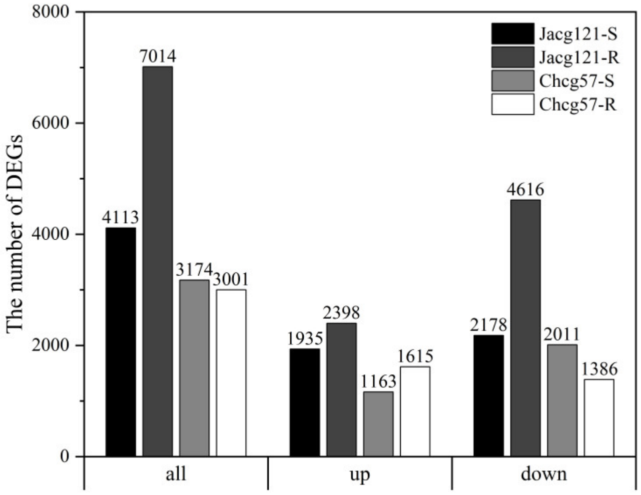

Supplemental Figure S1. The number of DEGs in drought-tolerant and drought-sensitive mycorrhizal seedlings under drought (field capacity 30%-35%) and well-watered (field capacity 85%-90%) treatments. S, shoot; R, root.

Supplemental Table S1. Geographic information of different ecotypes of *Cenococcum geophilum*.

Isolate	Isolate location
Jacg16	Miyasaki, Japan
Jacg21	Miyasaki, Japan
Jacg37	Numazu Senbonhama, Japan
Jacg81	Nagano, Japan
Jacg121	Tokyo, Japan
Jacg189	Tokyo, Japan
Jacg243	The Ryukyus University, Japan
Chcg57	Qinghai Province, China

Supplemental Table S2. ITS sequences of eight isolates of *Cenococcum geophilum*

Isolate	ITS sequences
Jacg16	AACCGTGAACTTCTAAACCTTTGACGATTGACTCATGTTGCCTCGGTGGGTT<br>CGCCCGCCAGAGGATACATCAAAACTCCTGTTTTAACGGTGTTGTCTGAGC<br>TACAAGCAACGAATCAAAACTTTCAACAACGGATCTCTTGGTTCTGGCATC<br>GATGAAGAACGCAGCGAAATGCGATAAGTAGTGTGAATTGCAGAATTCAGT<br>GAATCATCGAATCTTTGAACGCACATTGCGCCCCTTGGTATCCCGAGGGGCA<br>TGCCTGTTCGAGCGTCATTTCACCCCTCAAGCCTGGCTTGGTGTTGGGCGA<br>CGTCCTCTTTGGGGACGCGCCTCAAAACGCTCGGCGGCGTGGCACCGGCTT<br>TTAAGCGTAGCAGAATCTTATCTTTCGCTTTGAAAGTCGGGGCCATATCTGC<br>CGGAAGACCTACTCGCAAGGTTGACCTCGGATCAGGTAGG
Jacg21	GACCGCGAACTTTTAAACCTTTGATGATTGACTCATGTTGCCTCGGCGGGTT<br>CTCCCGCCAGAGGATGCATCAAAACTCCTGTTTTAACGGTGTTGTCTGAGCT<br>ACAAGCAACGAATCAAAACTTTCAACAACGGATCTCTTGGTTCTGGCATCG<br>ATGAAGAACGCAGCGAAATGCGATAAGTAGTGTGAATTGCAGAATTCAGTG<br>AATCATCGAATCTTTGAACGCACATTGCGCCCCTTGGTATCCCGAGGGGCAT<br>GCCTGTTCGAGCGTCATTTCACCACTCAAGCCTGGCTTGGTGTTGGGCGAC<br>GTCCCCTTCAGGGACGCGCCTCGAAACGCTCGGCGGCGTGGCACCGGCTTT<br>AAGCGTAGCAGAATCTTTCGCTTCAAAAGTCGGGGCCCCGTCTGCCGGAA<br>GACCTACTCGCAAGGTTGACCTCGGATCAGGCAGG
Jacg37	AACCGCGAACTTTTAAACCTTTGACGATTGACTCATGTTGCCTCGGCGGGT<br>TCTCTCGCCAGAGGATACATCAAAACTCCTGTTTTAACGGTGTTGTCTGAGC<br>TACAAGCAACGAATCAAAACTTTCAACAACGGATCTCTTGGTTCTGGCATC<br>GATGAAGAACGCAGCGAAATGCGATAAGTAGTGTGAATTGCAGAATTCAGT<br>GAATCATCGAATCTTTGAACGCACATTGCGCCCCTTGGTATCCCGAGGGGCA<br>TGCCTGTTCGAGCGTCATTTCACCACTCAAGCCTGGCTTGGTGTTGGGCGA<br>CGTCCCCTTCAGGGACGCGCCTCGAAACGCTCGGCGGCGTGGCACCGGCT<br>TTAAGCGTAGCAGAATCTTTCGCTTCAAAAGTTGGGGCCCCGTCTGCCGGA<br>AGACCTACTCGCAAGGTTGACCTCGGATCAGGCAGG
Jacg81	AACCGCGAACTTCTAAACCTTTGACGATTGACTCATGTTGCCTCGGCGGGT<br>TCGCCCGCCAGAGGATACATCAAAACTCCTGTTTTAACGGTGTTGTCTGAG<br>CTACAAGCAACGAATCAAAACTTTCAACAACGGATCTCTTGGTTCTGGCAT<br>CGATGAAGAACGCAGCGAAATGCGATAAGTAGTGTGAATTGCAGAATTCAG<br>TGAATCATCGAATCTTTGAACGCACATTGCGCCCCTTGGTATCCCGAGGGGC<br>ATACCTGTTCGAGCGTCATTTCACCACTCAAGCCTGGCTTGGTGTTGGGCG<br>ACGTCCCAAAGGGACGCGCCTCGAAACGCTCGGCGGTGTGGCACCGGCTT<br>TAAGCGTAGCAGAATCTTTCGCTTTAAAAGTCGGGGCCCCGTCTGCCGGAA<br>GACCTACTCGCAAGGTTGACCTCGGATCAGGTAGG
Jacg121	AACCGCGAACTTTTAAACCTTTGACGATTGACTCATGTTGCCTCGGCGGGT<br>TCTCTCGCCAGAGGATACATCAAAACTCCTGTTTTAACGGTGTTGTCTGAGC<br>TACAAGCAACGAATCAAAACTTTCAACAACGGATCTCTTGGTTCTGGCATC<br>GATGAAGAACGCAGCGAAATGCGATAAGTAGTGTGAATTGCAGAATTCAGT<br>GAATCATCGAATCTTTGAACGCACATTGCGCCCCTTGGTATCCCGAGGGGCA

	TGCCTGTTCGAGCGTCATTTCACCACTCAAGCCTGGCTTGGTGTTGGGGCG<br>ACGTCCCCTTCAGGGACGCGCCTCGAAACGCTCGGCGGCGTGGCACCGGC<br>TTTAAGCGTAGCAGAATCTTTCGCTTCAAAAGTTGGGGCCCCGTCTGCCGG<br>AAGACCTACTCGCAAGGTTGACCTCGGATCAGGCAGG
Jacg189	AACCGCGAACTTCTAAACCTTTGACGATTGACTCATGTTGCCTCGGCGGGT<br>TCTCCCGCCGGAGGATACATCAAAACTCCTGTTTTAACGGTGTTGTCTGAGC<br>TACAAGCAACGAATCAAAACTTTCAACAACGGATCTCTTGGTTCTGGCATC<br>GATGAAGAACGCAGCGAAATGCGATAAGTAGTGTGAATTGCAGAATTCAGT<br>GAATCATCGAATCTTTGAACGCACATTGCGCCCCTTGGTATCCCGAGGGGCA<br>TGCCTGTTCGAGCGTCATTTCACCACTCAAGCCTGGCTTGGTGTTGGGCGA<br>CGTCCCCTTCAGGGACGCGCCTCGAAACGCTCGGCGGCGTGGCACCGGCT<br>TTAAGCGTAGCAGAATCTTTCGCTTCAAAAGTCGGGGCCCCGTCTGCCGGA<br>AGACCTACTCGCAAGGTTGACCTCGGATCAGGTAGG
Jacg243	AACCGCGAACTTCTAAACCTTTGACGATTGACTCATGTTGCCTCGGCGGGT<br>CCTCCCGCCAGAGGATACATCAAAACTCCTGTTTTAACGGTGTTGTCTGAG<br>CTATAAGCAACGAATCAAAACTTTCAACAACGGATCTCTTGGTTCTGGCATC<br>GATGAAGAACGCAGCGAAATGCGATAAGTAGTGTGAATTGCAGAATTCAGT<br>GAATCATCGAATCTTTGAACGCACATTGCGCCCCTTGGTATCCCGAGGGGCA<br>TGCCTGTTCGAGCGTCATTTCACCACTCAAGCCTGGCTTGGTGTTGGGCGA<br>CGTCCCCTTTGGGGACGCGTCTCGAAACGCTTGGCGGCGTGGCACCGGCTT<br>TAAGCGTAGCAGAATATTTCGCTTTGAAAGTCGGGGCCCCGTCTGCCGGAA<br>GACCTACTCGCAAGGTTGACCTCGGATCAGGCAGG
Chcg57	AACCGCGAACTTCTAAACCTTTGACGATTGACTCATGTTGCCTCGGCAGGT<br>TCTCCCGCCAGAGGATACGTCAAAACTCCTGTTTTAACGGTGTTGTCTGAG<br>CTACAAGCAACGAATCAAAACTTTCAACAACGGATCTCTTGGTTCTGGCAT<br>CGATGAAGAACGCAGCGAAATGCGATAAGTAGTGTGAATTGCAGAATTCAG<br>TGAATCATCGAATCTTTGAACGCACATTGCGCCCCTTGGTATCCCGAGGGGC<br>ATGCCTGTTCGAGCGTCATTTCACCACTCAAGCCTGGCTTGGTGTTGGGCG<br>ACGTCCCCTTTGGGGACGCGCCTCGAAACGCTCGGCGGCGTGGCACCGGC<br>TTTAAGCGTAGCAGAATCTTTCGCTTCAAAAGTCGGGGCCCCGTCTGCCGG<br>AAGACCTACTCGCAAGGTTGACCTCGGATCAGGCAGG

Supplemental Table S3. Correlation coefficient matrix of single index of different ecotypes mycorrhizal seedlings after 7 days of drought stress (field capacity 30%-35%).

Index	SWR	RWC	Pn	Gs	Ci	Tr	CAT(S)	CAT(R)	POD(S)	POD(R)	SOD(S)	SOD(R)	MDA(S)	MDA(R)	PRO(S)	PRO(R)	SP(S)	SP(R)
SWR	1.000																	
RWC	0.963**	1.000																
Pn	0.672*	0.611	1.000															
Gs	0.801**	0.787*	0.717*	1.000														
Ci	0.670*	0.672*	0.121	0.697*	1.000													
Tr	0.582	0.576	0.670*	0.929**	0.500	1.000												
CAT(S)	-0.666	-0.567	-0.757*	-0.457	0.008	-0.353	1.000											
CAT(R)	-0.693*	-0.585	-0.735*	-0.360	0.038	-0.236	0.919**	1.000										
POD(S)	0.624	0.618	0.307	0.724*	0.586	0.711*	-0.396	-0.307	1.000									
POD(R)	0.572	0.559	0.443	0.740*	0.468	0.800**	-0.395	-0.333	0.943**	1.000								
SOD(S)	0.666	0.636	0.650	0.873**	0.451	0.903**	-0.479	-0.446	0.836**	0.865**	1.000							
SOD(R)	0.869**	0.822**	0.902**	0.842**	0.361	0.741*	-0.823**	-0.77*	0.563	0.584	0.785*	1.000						
MDA(S)	-0.761*	-0.707*	-0.415	-0.544	-0.490	-0.478	0.471	0.540	-0.520	-0.56	-0.481	-0.607	1.000					
MDA(R)	-0.892**	-0.837**	-0.743*	-0.819**	-0.486	-0.729*	0.725*	0.685*	-0.596	-0.596	-0.741*	-0.93**	0.819**	1.000				
Pro(S)	0.779*	0.780*	0.747*	0.953**	0.557	0.894**	-0.503	-0.438	0.661	0.655	0.902**	0.888**	-0.480	-0.84**	1.000			
Pro(R)	0.702*	0.726*	0.661	0.927**	0.555	0.882**	-0.411	-0.343	0.710*	0.687*	0.921**	0.809**	-0.355	-0.73*	0.979**	1.000		
SP(S)	0.611	0.600	0.648	0.864**	0.450	0.865**	-0.367	-0.345	0.660	0.661	0.940**	0.760*	-0.306	-0.678*	0.945**	0.970**	1.000	
SP(R)	0.812**	0.842**	0.476	0.709*	0.500	0.601	-0.545	-0.578	0.809**	0.713*	0.819**	0.755*	-0.580	-0.754*	0.789**	0.803**	0.755*	1.000

Note: SWC and RWC, shoot and root water contents; Pn, net photosynthetic rate; Gs, stomatal conductance; Ci, intercellular CO_2 concentration; Tr, transpiration rate; CAT, POD, and SOD, catalase, peroxidase, and superoxide dismutase activities; Pro, proline content; SP and SP, soluble protein contents; MDA, malondialdehyde (MDA) contents, S, shoot; R, root. * and ** mean significant differences at the levels of $P < 0.05$ and $P < 0.01$, respectively.

Supplemental Table S4. The index coefficient and proportion of comprehensive coefficient *CI* (comprehensive index).

Principle factors	CI_1	CI_2	CI_3
Eigen values	12.317	2.225	1.39
Contributive ratio	68.426	12.359	7.722
Cumulative contributive ratio	68.426	80.785	88.508
Eigenevector			
SWR	0.073	-0.092	-0.239
RWC	0.071	-0.051	-0.247
Pn	0.062	-0.179	0.272
Gs	0.076	0.102	0.041
Ci	0.047	0.213	-0.384
Tr	0.069	0.148	0.18
CAT(S)	-0.053	0.302	-0.075
CAT(R)	-0.05	0.334	-0.013
POD(S)	0.064	0.151	-0.119
POD(R)	0.064	0.131	-0.027
SOD(S)	0.074	0.104	0.163
SOD(R)	0.076	-0.135	0.095
MDA(S)	-0.055	0.096	0.391
MDA(R)	-0.074	0.106	0.092
PRO(S)	0.077	0.065	0.139
PRO(R)	0.073	0.125	0.175
SP(S)	0.069	0.127	0.257
SP(R)	0.071	0.013	-0.107

Note: *CI*, comprehensive index; SWC and RWC, shoot and root water contents; Pn, net photosynthetic rate; Gs, stomatal conductance; Ci, intercellular CO_2 concentration; Tr, transpiration rate; CAT, POD, and SOD, catalase, peroxidase, and superoxide dismutase activities; Pro, proline content; SP and SP, soluble protein contents; MDA, malondialdehyde (MDA) contents, S, shoot; R, root.

Supplemental Table S5. List of transcriptome data quality.

SampleID	Clean Reads	Mapped Reads	Mapped Ratio	Base Number
S121-DS1	22,950,191	6,871,129,962	45.74%	95.98%
S121-DS2	21,220,386	6,353,254,570	46.13%	95.88%
S121-DS3	22,095,266	6,616,185,438	46.06%	95.75%
S121-WW1	23,767,903	7,116,748,812	46.49%	95.79%
S121-WW2	19,695,861	5,893,797,682	46.61%	95.60%
S121-WW3	23,547,580	7,050,286,352	46.31%	95.51%
S57-DS1	21,223,106	6,356,316,998	45.55%	95.41%
S57-DS2	21,984,327	6,581,915,034	45.46%	95.51%
S57-DS3	21,435,830	6,417,880,884	45.95%	95.85%
S57-WW1	20,837,390	6,237,924,178	47.03%	95.66%
S57-WW2	20,113,617	6,022,856,442	46.58%	95.03%
S57-WW3	21,745,303	6,509,574,290	46.90%	95.92%
R121-DS1	21,972,497	6,575,392,614	46.40%	95.60%
R121-DS2	20,899,011	6,255,863,804	46.14%	95.35%
R121-DS3	20,166,029	6,036,205,202	45.46%	95.20%
R121-WW1	20,995,108	6,284,374,384	47.01%	95.42%
R121-WW2	20,880,607	6,251,771,070	47.28%	95.88%
R121-WW3	19,220,048	5,750,275,160	46.80%	95.82%
R57-DS1	21,598,880	6,462,707,120	46.63%	94.58%
R57-DS2	21,395,481	6,403,622,146	45.87%	94.98%
R57-DS3	20,113,218	6,013,574,536	46.56%	95.68%
R57-WW1	20,463,370	6,122,283,592	46.30%	95.82%
R57-WW2	21,059,284	6,303,590,750	46.46%	95.75%
R57-WW3	20,178,868	6,037,348,128	46.20%	95.42%

Note: S, Shoot; R, root; DS, drought stress; WW, well-watered;

Supplemental Table S6. Unigene length distribution statistics.

Length Range	Transcript	Unigene
300-500	14,575(21.12%)	10,201(31.85%)
500-1000	7,271(10.53%)	3,772(11.78%)
1000-2000	10,186(14.76%)	5,057(15.79%)
>2000	36,989(53.59%)	13,002(40.59%)
Total Number	69,021	32,032
Total Length	144,203,542	55,051,729
N50 Length	2,846	2,673
Mean Length	2089.27	1718.65

Supplemental Table S7. Sequences of primers used for RT-qPCR analysis.

Gene-ID	Primer orientation	Primer sequence
TRINITY_DN14578_c0_g1	Forward	TCTATCCCACGCCTCTTCCA
	Reverse	AGCCCTCAACGACATCAAGG
TRINITY_DN3930_c0_g1	Forward	CACGGTCACGCATTGACATC
	Reverse	GCCTTCGACCCAGCTCTATC
TRINITY_DN6096_c0_g1	Forward	ACGACTGGACAATACGCTCG
	Reverse	CCGCAGCCAGGATACATAGG
TRINITY_DN11509_c1_g1	Forward	ACGGTGGCATGCACAGAATA
	Reverse	TTCCAGTTGCCTCACTCGTC
TRINITY_DN12846_c0_g3	Forward	AGCTGATCCCGTTCCTTTGG
	Reverse	GGAATGCCTCCACCAGACTC
TRINITY_DN18667_c0_g1	Forward	AACAACGTCAACCTGCCTGA
	Reverse	GGTCGTTCAGTCTCTGGTGG
TRINITY_DN20940_c0_g2	Forward	AGGGACTCATGCAGACAATCT
	Reverse	GCATAAGGTGCCTCTCCGAC
TRINITY_DN40689_c0_g2	Forward	GCGCGCTCACATTTCTTCTG
	Reverse	TATTCACCAGCCCTCTGTGC
Aquaporin protein gene	Forward	CACCTTGCCACAATTCCTATCA
	Reverse	TCCAATGGTCATCCCAAACAC
